# Transcriptome Analysis Reveals Markers of Aberrantly Activated Innate Immunity in Vitiligo Lesional and Non-Lesional Skin

**DOI:** 10.1371/journal.pone.0051040

**Published:** 2012-12-10

**Authors:** Richard Yu, Raewyn Broady, Yuanshen Huang, Yang Wang, Jie Yu, Min Gao, Megan Levings, Shencai Wei, Shengquan Zhang, Aie Xu, Mingwan Su, Jan Dutz, Xuejun Zhang, Youwen Zhou

**Affiliations:** 1 Institute of Dermatology, Anhui Medical University, Hefei, China; 2 Department of Dermatology and Skin Science, University of British Columbia, Vancouver, Canada; 3 Department of Medicine, University of British Columbia, Vancouver, Canada; 4 Department of Microbiology and Immunology, University of British Columbia, Vancouver, Canada; 5 Department of Dermatology, The Third People's Hospital, Hangzhou, China; 6 Skin Tumor Group, British Columbia Cancer Agency, Vancouver, Canada; Dana-Farber Cancer Institute, United States of America

## Abstract

**Background:**

Vitiligo is characterized by the death of melanocytes in the skin. This is associated with the presence of T cell infiltrates in the lesional borders. However, at present, there is no detailed and systematic characterization on whether additional cellular or molecular changes are present inside vitiligo lesions. Further, it is unknown if the normal appearing non-lesional skin of vitiligo patients is in fact normal. The purpose of this study is to systematically characterize the molecular and cellular characteristics of the lesional and non-lesional skin of vitiligo patients.

**Methods and Materials:**

Paired lesional and non-lesional skin biopsies from twenty-three vitiligo patients and normal skin biopsies from sixteen healthy volunteers were obtained with informed consent. The following aspects were analyzed: (1) transcriptome changes present in vitiligo skin using DNA microarrays and qRT-PCR; (2) abnormal cellular infiltrates in vitiligo skin explant cultures using flow cytometry; and (3) distribution of the abnormal cellular infiltrates in vitiligo skin using immunofluorescence microscopy.

**Results:**

Compared with normal skin, vitiligo lesional skin contained 17 genes (mostly melanocyte-specific genes) whose expression was decreased or absent. In contrast, the relative expression of 13 genes was up-regulated. The up-regulated genes point to aberrant activity of the innate immune system, especially natural killer cells in vitiligo. Strikingly, the markers of heightened innate immune responses were also found to be up-regulated in the non-lesional skin of vitiligo patients.

**Conclusions and Clinical Implications:**

As the first systematic transcriptome characterization of the skin in vitiligo patients, this study revealed previously unknown molecular markers that strongly suggest aberrant innate immune activation in the microenvironment of vitiligo skin. Since these changes involve both lesional and non-lesional skin, our results suggest that therapies targeting the entire skin surface may improve treatment outcomes. Finally, this study revealed novel mediators that may facilitate future development of vitiligo therapies.

## Introduction

Affecting 0.5%–1% of population worldwide, vitiligo is an acquired pigmentation disorder in which melanocytes are destroyed, resulting in development of porcelain-white patches of skin [1], [2], [3]. Although non-fatal in nature, vitiligo causes severe negative psychosocial impact on the affected individuals, such as social stigmatization and decreased quality of life [4], [5].

The pathogenesis of vitiligo is largely unclear. There is evidence that vitiligo is an autoimmune disease [6], [7], [8], [9], [10], [11], [12], [13], [14], possibly involving additional factors such as oxidative stress [15] and genetic predisposition [6], [10], [11], [12], [13], [16], [17], [18]. The autoimmune hypothesis stems from the frequently observed association with other autoimmune diseases, such as hypothyroidism and diabetes [19], [20], [21], [22], [23]. This is further supported by the observation of T-lymphocyte infiltration in human vitiligo lesions and in mouse models [24] and the demonstration of melanocyte-specific antibodies in the blood of vitiligo patients. While these results suggest a role for the adaptive immune responses in melanocyte death [25], [26], [27], there are also early suggestions that innate, or natural, immunity in vitiligo is abnormal, as suggested by Jin *et al* [6], who demonstrated an association between vitiligo susceptibility and genetic changes in a critical innate immunity regulator gene, the NOD-like receptor 1 (NALP1), which has recently been confirmed by immunohistochemistry [28]. However, to date, direct evidence and independent confirmation of innate immunity in the skin of vitiligo patients have been sparse.

Previous vitiligo transcriptome analyses have focused on cultured melanocytes that were isolated from patients with vitiligo, demonstrating possible molecular abnormalities in vitiligo-patient's melanocytes [29], [30], [31], [32]. However, systematic detailed characterizations of vitiligo patient's skin, especially areas within well-established lesions away from the advancing border regions, as well as the normal appearing non-lesional areas, have not been performed previously, leaving unanswered the question of whether there are additional structural or molecular abnormalities present in vitiligo patient's skin other than the well-documented lack of melanocytes [33]. Answering this question may uncover additional clues to vitiligo pathogenesis and suggest novel approaches for future development of vitiligo therapies.

In this study, we performed a transcriptome analysis comparing gene expression profiles of three types of skin biopsies: (1) vitiligo lesional skin (LS); (2) normal appearing non-lesional skin (NLS) of vitiligo patients; and (3) normal skin of healthy volunteers (NS). This was followed by explant skin cultures and immunofluorescence analyses of the cellular infiltrates present in these skin biopsies. The results showed for the first time markers and cells of activated innate immune response not only in the lesional areas, but also in the normal appearing non-lesional areas of vitiligo patients' skin. This conclusion suggests that future therapeutic development needs to consider the role of innate immune activation in the affected as well as the unaffected areas of the skin in vitiligo patients. Therefore, treating the entire skin surface of vitiligo patients may have higher chances of achieving optimal therapeutic results than targeting individual lesions. Further, targeting innate immune activation may represent a promising approach for developing vitiligo therapies in the future.

## Materials and Methods

### Study Subjects and sample collection

Twenty-three subjects with vitiligo vulgaris and 16 healthy volunteers were recruited for this study (**Tables 1** **and** **2**). The diagnosis of vitiligo was based on acquired depigmentation of skin with typical symmetrical distribution on characteristic locations such as the torso, the extremities and the face. Wood's lamp was used to help establish the diagnosis. Paired 5 mm full-thickness punch biopsies were obtained from vitiligo lesional skin (LS, at least 2 cm inside the lesional border), non-lesional skin (NLS) located at least 2 cm outside of the border of the same lesion, or normal appearing skin on the non-involved contra-lateral side of the body. To minimize tissue heterogeneity due to anatomic variations, the biopsies were obtained from the torso and proximal extremities (proximal to the elbows and the knees) while avoiding the acral and facial locations. The biopsies were bisected into two equal portions, one placed in RNAlater solution (Invitrogen, Burlington, ON, Canada) for RNA extraction, while the other was immediately immersed in Histo-freeze medium and quickly frozen at a −80°C freezer for histological confirmation and structural characterization. For explant culture (see below) another 5 mm-punch biopsy was obtained from each subject (the lesional and non-lesional skin) and placed in the culture medium immediately.

**Table 1:** Demographics and clinical features of vitiligo subjects.

Subject No.	Sex	Ethnic Origin	Age (yrs)	Other Autoimmune Diseases	Family History of Autoimmune Diseases	1Disease Extent- BSA (%)	Biopsy Site	Types of Analysis
VIT1	M	Chinese	28	None	None	30	Elbow	MA, qPCR
VIT2	M	Caucasian	70	Myasthenia gravis	None	2	Arms	MA, qPCR
VIT3	M	S. Asian	54	None	None	5	Abd	MA, qPCR
VIT4	M	Chinese	20	None	Vitiligo	11	Legs	MA, qPCR
VIT5	F	S. Asian	35	None	None	10	Abd.	MA, qPCR
VIT6	M	S. Asian	75	None	None	3	Neck	MA, qPCR
VIT7	F	Chinese	21	None	None	5	Torso	MA, qPCR
VIT8	M	S. Asian	18	None	None	22	Abd.	MA, qPCR
VIT9	F	Chinese	71	None	None	2	Abd.	MA, qPCR
VIT10	M	Chinese	36	None	None	30	Abd.	MA, qPCR
VIT11	F	Chinese	33	Eczema	None	80	Flank	MA, qPCR
VIT12	F	Korean	28	None	None	20	Flank	MA, qPCR, IF
VIT13	F	S. Asian	57	None	None	6	Upper back	MA, qPCR, IF
VIT14	M	Chinese	51	None	Diabetes	2	Upper back	MA, qPCR, IF
VIT15	F	Caucasian	36	None	Vitiligo	3	Upper back	MA, qPCR, IF
VIT16	F	S. Asian	47	Hypothyroidism	None	3	Upper back	MA, qPCR, IF
VIT17	M	Chinese	26	None	None	1	Buttock	MA, qPCR, IF
VIT18	M	S Asian	71	None	None	10	Back	qPCR, Exp C, IF
VIT19	M	S Asian	65	None	None	7	Neck	qPCR, Exp C, IF
VIT20	M	Chinese	52	None	Diabetes	3	Neck	qPCR, Exp C, IF
VIT21	F	Chinese	53	None	None	25	Flank	qPCR, Exp C, IF
VIT22	F	Caucasian	32	None	None	5	Flank	qPCR, Exp C, IF
VIT23	M	Caucasian	27	None	Hypothyroidism, Vitiligo	10	Back	qPCR, Exp C, IF

Abbreviations: F = Female; M = Male; BSA = body surface area; Abd. = abdomen; VIT = subjects with vitiligo; MA = microarray; qPCR = quantitative polymerase chain reaction; Exp C = Explant skin cultures; IF = immunofluorescence.

1 Percent body surface area involvement are estimations using estimated number of palm areas covered by the white patches of skin, with each palm area representing as 1% body surface area.

**Table 2:** Demographics and clinical features of normal control subjects.

Subject No.	Sex	Ethnic Origin	Age (yrs)	Other Autoimmune Diseases	Family History of Autoimmune Diseases	1Disease Extent- BSA (%)	Biopsy Site	Types of Analysis
NS1	M	Chinese	48	None	NA	NA	Back	MA, qPCR
NS2	F	S Asian	55	None	NA	NA	Back	MA, qPCR
NS3	M	Chinese	43	None	NA	NA	buttock	MA, qPCR
NS4	M	Chinese	70	None	NA	NA	Back	MA, qPCR
NS5	F	Caucasian	28	None	NA	NA	Abdo	MA, qPCR
NS6	M	Caucasian	48	None	NA	NA	Chest	MA, qPCR
NS7	M	Caucasian	65	None	NA	NA	Chest	MA, qPCR
NS8	F	Chinese	49	None	NA	NA	Abdo	MA, qPCR
NS9	M	Chinese	78	None	NA	NA	Thigh	MA, qPCR
NS10	F	S Asian	50	None	NA	NA	Abdo	MA, qPCR
NS11	F	Caucasian	25	None	NA	NA	Back	MA, qPCR, IF
NS12	M	S Asian	34	None	NA	NA	Neck	MA, qPCR, Exp C, IF
NS13	F	Caucasian	36	None	NA	NA	Back	MA, qPCR, Exp C, IF
NS14	M	S Asian	31	None	NA	NA	Abdo	MA, qPCR, Exp C, IF
NS15	F	Chinese	26	None	NA	NA	Flank	MA, qPCR, Exp C, IF
NS16	M	Caucasian	22	None	NA	NA	buttock	MA, qPCR, Exp C, IF

Abbreviations: F = Female; M = Male; BSA = body surface area; Abd. = abdomen; NS = normal skin from healthy volunteers; NA = not available; MA = microarray; qPCR = quantitative polymerase chain reaction; Exp C = Explant skin cultures; IF = immunofluorescence.

1 Percent body surface area involvement are estimations using estimated number of palm areas covered by the white patches of skin, with each palm area representing as 1% body surface area.

The study was approved by the Ethical Review Board of the University of British Columbia (Certificate Number C98-0493) in accordance with the contents of the Declaration of Helsinki, and by the Institutional Review Board of the University of British Columbia. Collection of skin biopsies was undertaken after the subjects have signed the informed consent.

### RNA extraction

Skin samples were trimmed to remove visible adipose tissue and homogenized in Trizol (Invitrogen, Burlington, ON, Canada) using the tissue homogenizer (Model 398; Biospec Products Inc, Bartlesville, OK, USA). RNeasy Fibrous Tissue Mini Kit (Qiagen, Valencia, CA, USA) was used to extract total cellular RNA according to the manufacturer's protocol. The quality of the RNA was assessed by the Agilent Bioanalyzer 2100 (Agilent Technologies, Santa Clara, CA, USA); and the concentration of the RNA samples was determined by the Nanodrop 1000 spectrophotometer (Thermo Scientific, Ottawa, ON, CA).

### Transcriptome analysis using DNA microarrays and molecular pathway analysis

For the initial screening of gene expression differences in vitiligo skin, RNA from 17 pairs of full-thickness vitiligo skin and 16 normal skin biopsies were used in two-colored DNA microarray analysis following a protocol we previously described [34], [35], [36].. Briefly, total cellular RNA (500 ng) was reverse-transcribed into cDNA and linearly amplified by *in vitro* transcription in the presence of fluorescent-labeled CTP using the Low RNA Input Linear Amplification Kit, PLUS, Two-Color from Agilent following the manufacturer's instructions. Each microarray was hybridized with 825 ng of amplified cDNA labelled with Cy5 (each individual RNA sample) or Cy3 (pooled control skin RNA from 16 individual donors) at a specific activity between 8 and 15 pmol/µg. Hybridizations were performed on Whole Human Genome Oligo microarrays (G4112F, Agilent Technologies, Santa Clara, CA, USA), comprising 41,059 60-nt oligonucleotide probes, mostly represented as single spots. Image scanning was performed with the Agilent DNA Microarray Scanner and quantified using Agilent's Feature Extraction software. The results were imported and analyzed with the GeneSpring GX 7.3 software (Agilent Technologies, Santa Clara, CA, USA) for statistical computing and visualization. Data normalization was performed within and across the arrays using per gene, per chip normalization according to the Agilent recommendation. To detect the differentially expressed genes between vitiligo LS and NLS and NS, non-parametric Mann-Whitney U tests were used based on group analysis. The genes were ranked according to their false discovery rate-adjusted p-values (with cut off set at <0.05 after Bonferroni corrections). The threshold of expression differences was set at a 2.0-fold increase or decrease in gene expression levels as compared with NS.

Pathway analysis on the differentially expressed genes in vitiligo skin was performed using Database for Annotation, Visualization and Integrated Discovery (DAVID) Bioinformatics Resources 6.7, which is a standard bioinformatics tool for functional analysis of large gene list derived from high-throughput genomic scanning [37], [38]. It systematically maps a large number of genes in a list to the associated biological annotation terms (e.g. GO Terms or Pathways), and then examine the statistical significance of the gene enrichment by comparing the outcome to the reference controls [38]. Vitiligo differentially-expressed gene lists were also mapped to the Kyoto Encyclopedia of Genes and Genomes (KEGG) pathway database. All annotated pathways were ranked by enrichment score and Benjamini adjusted p values.

### Confirmation of gene expression changes by quantitative polymerase chain reaction

The candidate differentially-expressed genes that were identified by transcriptome analysis were verified by quantitative real time polymerase chain reaction (qRT-PCR) according to methods we previous reported [34], [39] on all samples used in the transcriptome analysis plus 6 additional pairs of vitiligo samples. Briefly, total cellular RNA was extracted using the RNeasy Mini Kit (Qiagen, Mississauga, ON, Canada) following manufacturer's protocol. The isolated RNA was reverse transcribed using random primers and SuperScript III reverse transcriptase (Invitrogen, Burlington, ON, Canada). Real-time PCR was performed on a DNA Engine Opticon™ System (Bio-Rad Laboratories, Mississauga, ON, Canada) using the SYBR® Green method and analyzed with glyceraldehyde-3-phosphate dehydrogenase (GAPDH) as the internal control. The statistical significance of the gene expression differences was calculated using paired two-tailed Student t tests (for comparisons between LS and NLS) or two-tailed non-parametric Whitney U tests (between vitiligo skin biopsies and healthy control skin biopsies).

### Explant culture of natural killer cells

Immune cells from the skin biopsies of 6 vitiligo patients and 5 healthy individuals were isolated and cultured as previously described [40], [41]. Briefly, Cell-foam matrices (Cellsciences Pte Ltd, Singapore) were treated with rat tail collagen I (BD Biosciences, Bedford, MA, USA) and served as three-dimensional scaffolds that separate dermal fibroblasts and skin-resident lymphocytes. The skin explants were minced and placed on the surface of the matrices and cultured in 12-well 0.4 mm pore size polyester trans-well culture plates (Corning, Corning, NY, USA) in the presence of 25 ng/ml IL-2 and 20 ng/ml IL-15 (R&D systems, Minneapolis, MN, USA) in IMDM (Stemcell Technologies, Vancouver, BC, Canada). The cultures were supplemented with 10% heat-inactivated fetal bovine serum (Hyclone; Thermo Scientific, Ottawa, ON, CA), penicillin and streptomycin (Sigma-Aldrich, Oakville, ON, Canada). The cells that have migrated out of the explants were analyzed after 3 weeks by flow cytometry.

### Analysis of natural killer cells by flow cytometry

Flow cytometry of the immune cells migrating out of the skin biopsies in culture dishes was performed using fluorophore-conjugated antibodies against T cell receptors and natural killer (NK) cell markers. Cells were labeled with Fixable Viability Stain 450 and antibodies against CD3 APC (Ms IgG_1_: SK7), CD56 FITC (Ms IgG_2b_: NCAM16.2), Ki67 PE (Ms IgG_1_: B56) and granzyme B APC (Ms IgG_1_: GB11) (BD Biosciences, Bedford, MA, USA), where NK cells were distinguished from the rest of the lymphocytes via positive expression of CD56 and negative expression of CD3. The samples were analyzed on a BD FACSCanto flow cytometer (BD Biosciences, Bedford, MA, USA) and data was analyzed with FCS Express Pro Software Version 3 (De Novo Software, Los Angeles, CA, USA). A total of 10,000 events were collected for each sample, and live cells (positive for the viability stain) were analyzed for the proportion of CD3-CD56+ NK cells. These cells were further gated to obtain the proportion of NK cells expressing granzyme B.

### Assessment of natural killer cells in skin biopsies by immunofluorescence microscopy

Biopsies were embedded in Histo-freeze medium upon collection and transferred to −80°C for storage. Sections of 14 to 20 µm in thickness were cut using a cryostat and subjected to standard immunofluorescence staining protocol. Briefly, the sections were fixed in 4% paraformaldehyde (Sigma-Aldrich, Oakville, ON, Canada) at 4°C for 20 min. For permeabilization, 0.3% Triton X (Sigma-Aldrich, Oakville, ON, Canada) was applied to the sections for 10 min at 4°C. After blocking in 10% normal goat serum (Sigma-Aldrich, Oakville, ON, Canada) for 60 min at room temperature, the sections were incubated overnight in mouse monoclonal anti-human NKG2D (Abcam, Cambridge, MA, USA), which labels NK cells; rabbit polyclonal anti-human CD3 (Dako, Burlington, ON, CA), which labels T lymphocytes; or rabbit polyclonal anti-human Melan-A/MART1 (Sigma-Aldrich, Oakville, ON, Canada), which identifies the melanocytes. Then, the slides were treated with Alexa Fluor® 594 goat anti-mouse IgG and Alexa Fluor® 488 goat anti-rabbit IgG (Invitrogen, Burlington, ON, Canada). Finally, the sections were counter-stained with DAPI (Sigma-Aldrich, Oakville, ON, CA) and mounted in Gold anti-fade medium (Invitrogen, Burlington, ON, CA). The slides were visualized with a Zeiss Axiovert 200M inverted fluorescence microscope (Zeiss, Toronto, ON, Canada). Image processing and quantification were performed with AxioVision Rel. 4.6 software (Zeiss, Toronto, ON, Canada) using the interactive measurement module. Quantification of cells was performed across entire tissue sections, with the resulting data expressed as the mean number of cells per 400× field of view over a range of 20–32 fields of view depending on the size of the specimens.

### Statistical analysis

The transcriptome analyses were performed using GeneSpring GX 7.3 software (Agilent Technologies, Santa Clara, CA, USA). Additional statistical analyses were performed with GraphPad Prism software (GraphPad Software, Inc, La Jolla, CA, USA). Non-parametric Mann-Whitney U tests were used to compare the difference in gene expression and the quantity of natural killer cells between vitiligo skin and the normal healthy skin.

## Results

### 1. Demographics and clinical characterization of study subjects

All study subjects had the diagnosis of vitiligo vulgaris, with a mean of 17.8% (range: 1% to 80%) body skin area depigmented (**Table 1**). The subjects were from the Vancouver General Hospital Skin Care Centre. There were 11 East Asians (Chinese and Koreans), 8 South Asians (Indians and Pakistanis) and 4 Caucasians. Twelve were males. The average age was 43.7 years (Range 18–75). Eight of the 23 subjects (34.8%) had a personal or family history of autoimmune diseases such as thyroid diseases or vitiligo. The healthy volunteers were of similar gender and ethnic composition as the vitiligo subjects but without vitiligo or other skin diseases.

### 2. Gene expression changes in vitiligo lesional skin

**Figure 1:**
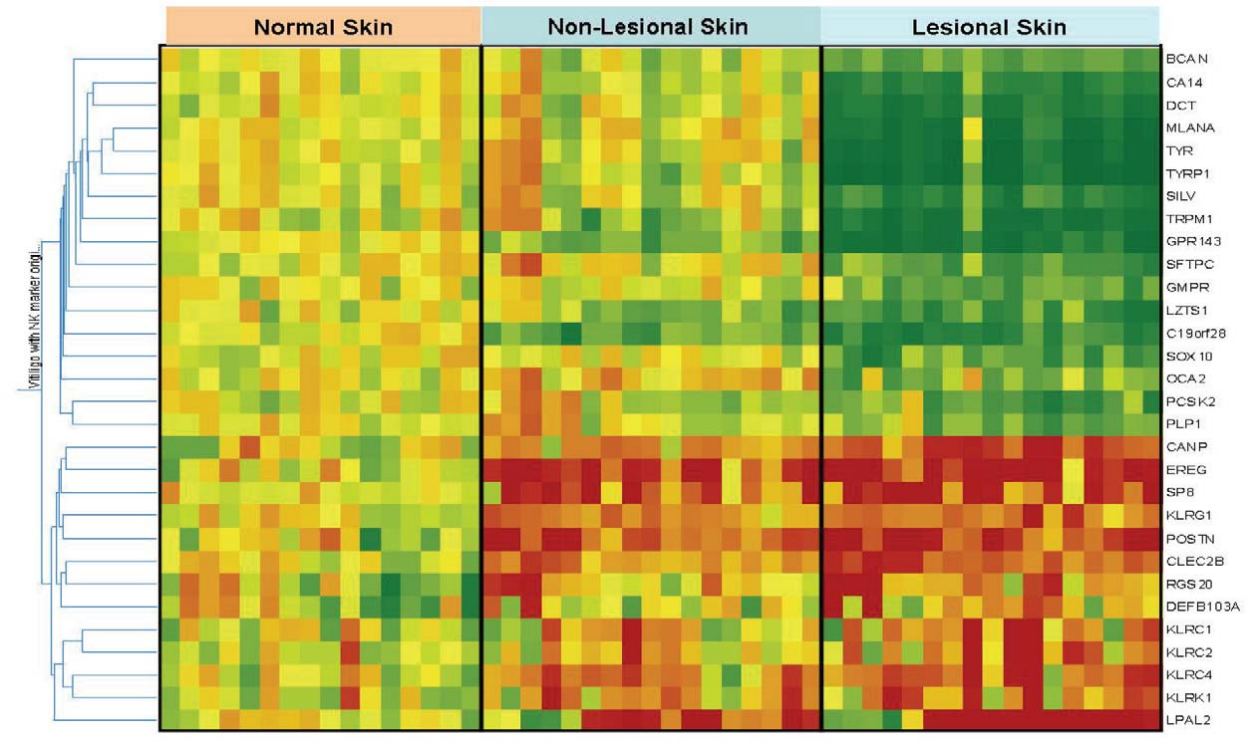
Transcriptome Analysis of Vitiligo and Normal Skin Biopsies. A heat map is constructed by Gene Spring software (see methods) comparing the relative expression levels of the 30 significantly altered genes in vitiligo skin. Depicted are the expression levels of these genes in individual samples relative to their corresponding expression reference levels, which are the averages of expression in the 16 normal skin biopsies. ***Red squares***: Genes with up-regulation in that sample compared with normal skin of healthy volunteers. ***Green squares***: Genes with down-regulation in that specific sample compared with normal skin of healthy volunteers. ***Yellow squares***: no significant change between the sample and the normal skin of healthy volunteers.

We first examined gene expression changes in vitiligo lesional skin (LS) using the skin from healthy subjects as the controls (**Figure 1** and **Tables 3** **and** **4**). Based on microarray analysis, there were 30 genes with significant expression changes (>2 fold up- or down- regulation, p<0.05, non-parametric Mann-Whitney U tests, with Bonferroni correction for multiple-testing). Of these, 17 were down-regulated (**Table 3**) and 13 up-regulated (**Table 4**) in vitiligo LS compared with healthy normal skin (NS), which are further confirmed with qRT-PCR analysis of additional samples. As expected, most of the down-regulated genes encode lineage markers or functional components of melanocytes, including TYRP1, TYR, Melan-A, and SILV. Several neuron-related genes, including PLP1, which encodes the major myelin associated protein (a specific marker for the Schwann cells) were also decreased. Among the up-regulated genes, the vast majority encode innate immunity regulators, such as β-defensin and CLEC2B (an activating ligand for natural killer cells that is primarily expressed by macrophages), as well as multiple activation markers of the NK cells (another important player in innate immunity). Among the other notable up-regulated genes, CANP codes for calpains, a family of proteases strongly associated with oxidative stress [42], which may participate in melanocyte destruction in vitiligo [15]; POSTN codes for periostin, a protein involved in tissue injury, repair and remodeling [43], although its role in vitiligo is unclear at present.

**Table 3:** Down-regulated genes in vitiligo skin.

Gene	Chrom	1Microarray			2qRT-PCR			Function
	osome	LS/NS	NLS/NS	LS/NLS	LS/NS	NLS/NS	LS/NLS	
**TYRP1**	9	0.06 *	0.97	0.06 *	0.02 *	1.34	0.01 *	Melanogenesis
**TYR**	11	0.08 *	1.09	0.07 *	0.02 *	1.25	0.02 *	Melanogenesis
**MLANA**	9	0.13 *	1.08	0.12 *	0.04 *	1.44 *	0.02 *	Melanogenesis
**TRPM1**	15	0.11 *	0.89	0.12 *	0.07 *	1.82 *	0.04 *	Melanogenesis and signal transduction
**DCT**	13	0.13 *	0.90	0.14 *	0.01 *	0.51 *	0.02 *	Melanogenesis
**PMEL**	12	0.21 *	1.02	0.21 *	0.22 *	2.16 *	0.10 *	Melanogenesis
**CA14**	1	0.21 *	0.89	0.24 *	0.33 *	1.98 *	0.17 *	Physiological processes
**SFTPC**	8	0.35 *	1.21	0.29 *	0.14 *	2.53 *	0.06 *	Surfactant
**SOX10**	22	0.31 *	0.80	0.39 *	0.02 *	0.20 *	0.08 *	Stem cell development
**OCA2**	15	0.58 *	1.44	0.40 *	0.46 *	1.06	0.43 *	Melanogenesis
**PCSK2**	20	0.36 *	0.89	0.40 *	0.18 *	1.05	0.17 *	Hormone synthesis
**PLP1**	X	0.50 *	1.14	0.44 *	0.23 *	0.57 *	0.41 *	Myelin sheath protein
**GMPR**	6	0.40 *	0.84	0.48 *	0.25 *	0.69 *	0.37 *	Nucleoside reductase
**BCAN**	1	0.37 *	0.78	0.47 *	0.15 *	0.87	0.17 *	Cell motility
**LZTS1**	8	0.31 *	0.63 *	0.49 *	0.10 *	0.44 *	0.23 *	Tumor suppressor
**GPR143**	X	0.10 *	0.51 *	0.20 *	0.09 *	0.76 *	0.12 *	Melanogenesis and signal transduction
**C19orf28**	19	0.22 *	0.44 *	0.50 *	0.21 *	0.87	0.25 *	Unknown

1 Ratios (LS/NS, NLS/NS, LS/NLS) are calculated based on the mean expression levels in 17 vitiligo skin and the mean expression levels in 16 normal skin. * p<0.05 after Bonferroni correction (Whitney U tests).

2 Ratios (LS/NS, NLS/NS, LS/NLS) are calculated based on the mean expression levels in 23 vitiligo skin and the mean expression levels in 16 normal skin. * p<0.05 (Whitney U tests).

**Table 4:** Up-regulated genes in vitiligo skin.

Gene	Chrom	1Microarray			2qRT-PCR			Function
	osome	LS/NS	NLS/NS	LS/NLS	LS/NS	NLS/NS	LS/NLS	
**KLRC1**	12	6.27 *	1.71	3.67 *	7.39 *	4.71 *	1.57 *	Natural killer cell receptor
**KLRC2**	12	5.88 *	1.44	4.08 *	8.55 *	9.26 *	0.92	Natural killer cell activating receptor
**NKG2D (KLRK1)**	12	2.48 *	2.02 *	1.23	6.07 *	5.98 *	1.01	Natural killer cell activating receptor
**KLRG1**	12	2.46 *	2.05 *	1.20	3.92 *	2.86 *	1.37	Natural killer cell receptor
**KLRC4**	12	3.91 *	2.27 *	1.72	7.91 *	5.37 *	1.47 *	Natural killer cell receptor
**LPAL2**	6	6.37 *	3.28 *	1.94 *	7.65 *	5.14 *	1.49 *	Pseudogene
**CANP**	11	3.56 *	1.92 *	1.85 *	1.62 *	1.69 *	0.96	Oxidative stress
**DEFB103A**	8	2.27 *	1.38	1.64	11.62 *	9.27 *	1.25	Innate immunity
**CLEC2B**	12	3.18 *	2.01 *	1.58	9.81 *	11.13 *	0.88	Ligand for natural killer cell receptor
**SP8**	7	6.48 *	4.15 *	1.56	5.53 *	8.15 *	0.68 *	Transcription factor
**POSTN**	13	5.70 *	3.68 *	1.55	4.23 *	2.76 *	1.53 *	Tissue injury and repair
**RGS20**	8	3.10 *	2.02 *	1.53	10.31 *	6.11 *	1.69 *	Signal transduction
**EREG**	4	7.46 *	5.37 *	1.39	7.09 *	6.74 *	1.05	Epidermal growth factor

1 Ratios (LS/NS, NLS/NS, LS/NLS) are calculated based on the mean expression levels in 17 vitiligo skin and the mean expression levels in 16 normal skin. * p<0.05 after Bonferroni correction (Whitney U tests).

2 Ratios (LS/NS, NLS/NS, LS/NLS) are calculated based on the mean expression levels in 23 vitiligo skin and the mean expression levels in 16 normal skin. * p<0.05 (Whitney U tests).

Pathway analysis with Database for Annotation, Visualization and Integrated Discovery (DAVID) Bioinformatics Resources 6.7 [37], [38] showed that the differentially expressed genes in vitiligo lesional skin point to four enriched pathways: (1) tyrosine metabolism; (2) melanin biosynthesis, (3) natural killer cell cytotoxicity, and (4) antigen processing (**Table 5**). This further highlights the potential importance of innate immunity and natural killer (NK) cells in vitiligo skin.

**Table 5:** Enriched molecular pathways in vitiligo differentially expressed genes*.

Pathway	p-Value	p-value (Benjamini adjusted)
Tyrosine metabolism	0.0025	0.023
Antigen processing and presentation	0.0088	0.039
Melanogenesis	0.012	0.037
Natural killer cell mediated cytotoxicity	0.022	0.048

* Pathway analysis on the differentially expressed genes in vitiligo skin was performed using Database for Annotation, Visualization and Integrated Discovery (DAVID) Bioinformatics Resources 6.7 [37], [38]. All annotated pathways were ranked by enrichment score and Benjamini adjusted p value.

### 3. Gene expression changes in non-lesional skin of vitiligo patients

Further comparisons were made between the NLS of vitiligo patients and NS from healthy subjects. As shown in **Figure 1** and **Tables 3** **and** **4**, most of the genes whose expression was down-regulated in vitiligo LS (including the melanocyte markers) showed no such change in NLS. In contrast, the expression levels of most up-regulated genes in vitiligo LS, including all of the innate immunity activation markers, were also increased in the normal appearing NLS skin of vitiligo patients, suggesting that the activation of the innate immunity is not just limited to the LS. Rather, innate immunity activation may be present throughout the entire skin surface of vitiligo patients.

### 4. Analysis and quantification of natural killer cells in the lesional and non-lesional skin of vitiligo patients

**Figure 2:**
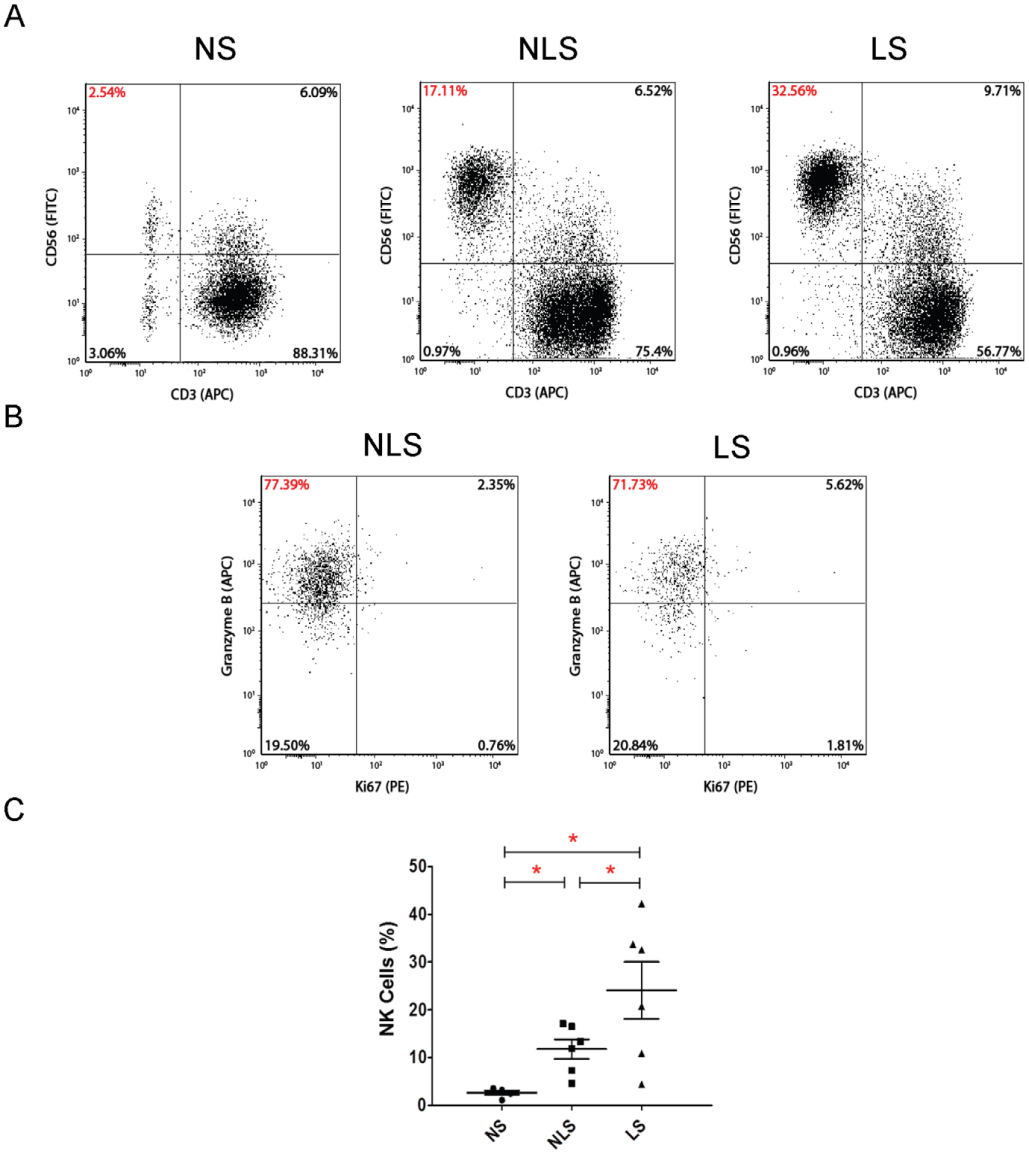
Explant culture analysis of natural killer cell infiltrates in biopsies of vitiligo lesional and non-lesional skin. Natural killer (NK) cells from 6 pairs of vitiligo skin explants (lesional and non-lesional) and 5 normal skin explants were cultured on Cellfoam matrices (see methods section) and analyzed using flow cytometry, with the gate set on total live cells **A:** Skin-resident CD56^bright^ CD3-ve NK cells in normal control skin, vitiligo non-lesional skin and lesional skin by scatter plot. **B:** Further gating on the CD56^bright^ CD3-ve cells revealed that majority of the NK cells in vitiligo skin were granzyme B-positive. **C:** Dot plot of all samples analyzed for CD56^bright^ CD3- natural killer cells. The difference in the proportion of resident natural killer cells between normal skin and the respective vitiligo non-lesional and lesional skin is statistically significant (p = 0.0043; mean ± SEM). Comparisons between the respective groups are indicated in the figure by lines with an asterisk (*) denoting statistical significance (p<0.05). Abbreviations: NS: normal skin; NLS: non-lesional skin; LS: lesional-skin.

Since the gene expression analyses revealed markedly increased NK cell markers in NLS and LS of vitiligo patients, we speculated that the skin of vitiligo subjects contain abnormal infiltration of NK cells. To test this speculation, skin resident immune cells were isolated from cultured vitiligo skin explants [40], [41] and analysed for cellular compositions and activation status using antibodies against CD3 (a pan T cell marker), CD56 (a specific natural killer cell marker) and granzyme B (a cytotoxicity marker for NK cells). Skin from healthy volunteers served as the controls. The explant culture method has previously been shown to accurately reflect the *in situ* immune cell compositions in the tissues [41]. As shown in **Figure 2A and C**, NS contained only a small percentage of NK cells (less than 5% of total immune cells present). In contrast, in the skin obtained from vitiligo patients, there was a significant increase in the proportion of NK cells not only in the LS (24%), but also in the normal-appearing NLS (12%). In addition, the NK cells cultured from vitiligo skin explants exhibited high levels of the serine protease, granzyme B (**Figure 2B**), indicating that these cells are highly active and capable of exerting cytotoxicity on the target cells by contact [**44**].

**Figure 3:**
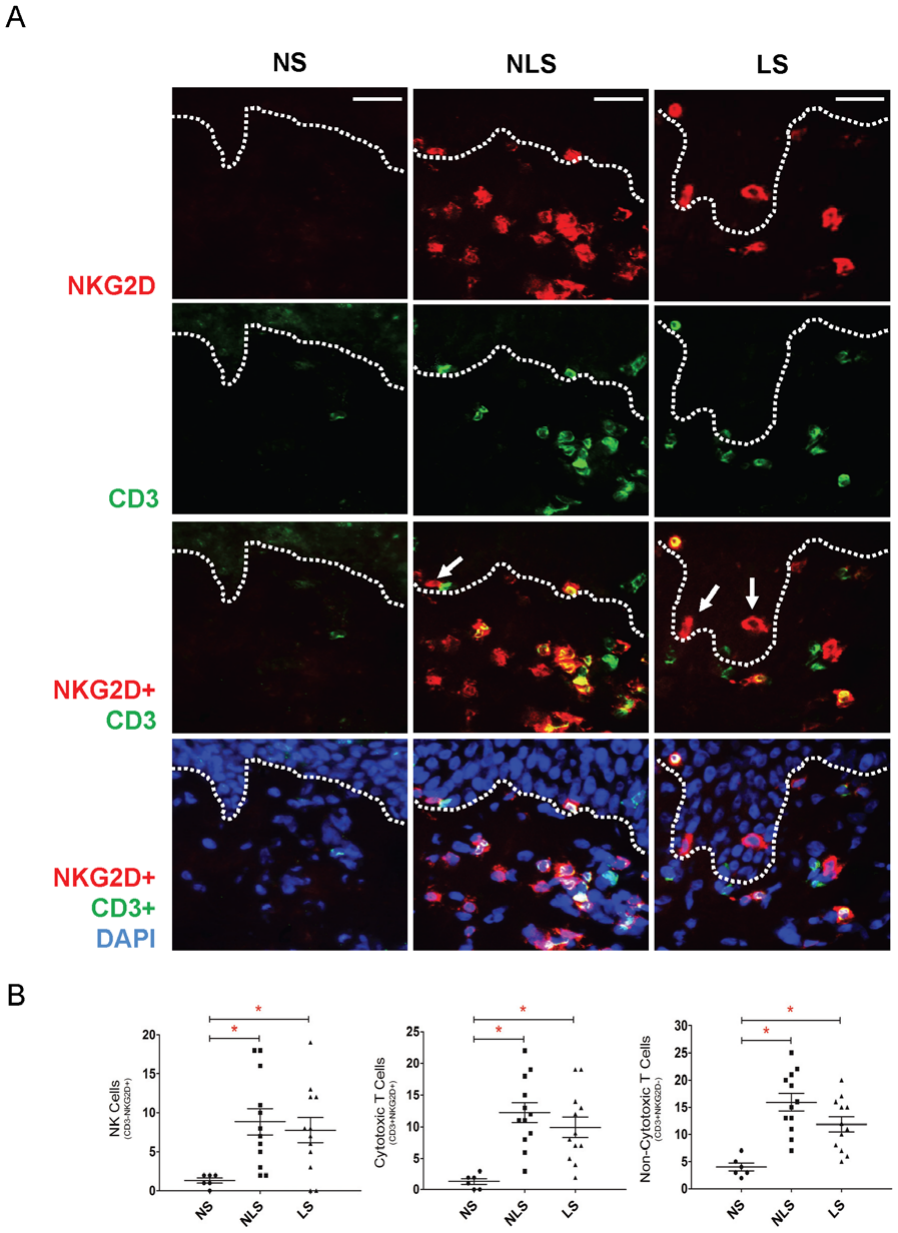
Distribution of natural killer cells and cytotoxic T cells in vitiligo lesional and non-lesional skin. Skin biopsies taken from 12 vitiligo patients and 6 normal individuals were subjected to immunofluorescence analysis of natural killer (NK) cells. **A:** Micrographs showing natural killer cells (CD3−/NKG2D+) (red) present in vitiligo lesional and non-lesional skin but absent from the normal skin of healthy volunteers. Some NK cells are in close proximity to the basal epidermal layer where melanocytes reside (arrows). In addition, increased numbers of cytotoxic T cells (CD3+/NKG2D+) (yellow: co-localization of red and green) as well as non-cytotoxic T cells (CD3+/NKG2D−) (green) were also found in both vitiligo peri-lesional and lesional skin. **B:** Quantification of cells demonstrates a statistically significant increase in NK cells, cytotoxic T cells and non-cytotoxic T cells in vitiligo non-lesional skin (p = 0.0021, 0.0015, 0.001; mean ± SEM) and lesional skin (p = 0.021, 0.0017, 0.0023; mean ± SEM) as compared with normal skin. Color keys: ***Green***: CD3 (a pan-T cell marker); ***Red***: NKG2D (NK cell activation receptor); and ***blue***: DAPI (nuclear stain). Comparisons between the respective groups are indicated in the figure by lines with an asterisk (*) denoting statistical significance (p<0.05). Abbreviations: NS: normal skin; NLS: non-lesional skin; LS: lesional-skin. Magnification: 400×; scale bar: 20 µm.

To further confirm the increased NK cell infiltration within the skin of vitiligo patients, we next examined the biopsies by immunofluorescence microscopy. As shown in **Figure 3A and 3B**, both lesional and non-lesional vitiligo skin harboured markedly increased number of NK cells as demonstrated by the presence of CD3-NKG2D+ cells as compared to the control skin of healthy individuals. Some of these CD3-NKG2D+ NK cells were in the epidermis and/or and were in close proximity to the basal layer (arrows), where cutaneous melanocytes normally reside. In addition, increased numbers of CD3+ NKG2D+ (cytotoxic T cells) cells and CD3+ NKG2D− cells (non-cytotoxic T cells) were also observed in vitiligo skin, which is consistent with previous findings **[28]**, **[45]**.

## Discussion

This study examined the gene expression profiles of skin tissues from patients with vitiligo using skin biopsies of healthy individuals as the controls. A potential technical challenge of the current study arises from the fact that melanocytes only account for a small proportion of total cells in the full thickness skin biopsies, which theoretically makes it difficult to detect melanocyte-related gene changes. The fact that the down-regulated genes identified by this non-cell-targeted analysis included the melanocyte markers among the most significantly altered genes demonstrated the robustness of the current approach in picking up gene expression changes even if these changes only account for a small fraction of the total cells present in the skin biopsy tissues.

Most of the down-regulation of melanocyte-related genes (such as Tyr, TYRP1) in the well-established vitiligo lesional skin (LS) most likely is the result of melanocyte death in the lesional skin. The expression down-regulation of several other genes, including LZTS1, GPR143 and C19orf28 does not have clear explanation at the present. It is possible that these genes are also melanocyte-expressed genes although this remains to be clearly established. The down regulation of SOX10 and PLP1 in vitiligo lesional skin may also be partially explained by the fact that they can be found in melanocytes [46], [47]. However, they are also found in Schwann cells [48], raising the possibility that these cells may be damaged although direct results are lacking at present. This possibility is consistent with previous reports of degenerative changes in Schwann cells in vitiligo skin as revealed by electron microscopy [49], [50].

The significance of the down-regulation of some melanocyte related genes (such as DCT, SOX10 and PLP1) in vitiligo non-lesional skin as compared with normal skin of healthy individual is not entirely clear, but may represent subclinical melanocyte damage even in the lack of overt death of melanocytes. Alternatively, they may reflect inherent abnormalities present in the vitiligo individuals' melanocytes, as have been suggested to be present by the observation of gene expression abnormalities in purified melanocytes from vitiligo individuals [29], [30], [31], [32].

The unique findings from the current study include notably the discovery of the up-regulation of multiple genes of the killer cell lectin-like receptor (KLR) family including multiple NK cell activation markers such as KLRK1 (also known as NKG2D), KLRC2 and KLRC4 [51], [52], [53]. NK cells are an important component of the innate immune system. Although the most notable role of NK cells is in the defense against bacterial, viral and parasitic infections, there is strong evidence that NK cells play an important role in the initiation and/or perpetuation of autoimmune diseases [54], [55]. In particular, elevated numbers of CD56^bright^ NK cells are found in the blood and target lesions of patients with systemic lupus erythematosus and rheumatoid arthritis [56], [57], [58]. NK cell receptors and their ligands are also implicated in autoimmune cholangitis, multiple sclerosis and psoriasis [59], [60], [61], [62].

Previous studies have shown an increase in the number of circulating NK cells in the blood of vitiligo patients [63], [64], [65], [66], [67]. Our present work has demonstrated the infiltration of NK cells in the skin microenvironment of melanocytes in both the lesional and non-lesional area of the skin of vitiligo patients. Further, these cells express high levels of granzyme B, which is characteristic of activated NK cells [44], [68], [69], [70]. In addition to the commonly attributed cytotoxic role via activation of the caspase pathway, granzyme B has been implicated in the generation and presentation of auto-antigens [71].

The fact that increased NK cell infiltration was observed in the normal-appearing NLS of vitiligo patients may indicate an unfavorable generalized melanocyte-microenvironment in the skin compartment for the melanocyte to survive. However, what gives rise to the NK cell/innate immunity activation in vitiligo skin is not clear. It has been documented that NK cells respond to cellular signals released by cells under stress [72]. In particular, it has been shown previously that ligands for the NKG2D receptor, which are MHC class I-like proteins in the ULBP and MIC families, can be induced in stressed cells and via the inflammatory cascade, and can result in marked alteration in the immune microenvironment [51], [73]. One of the well-known ligands in the ULBP family—ULBP2, has been up-regulated in vitiligo skin in our microarray analysis as well (around 1.5 folds increase in vitiligo skin), although the data did not meet our strict cut-off criteria (at 2 fold). Further, our transcriptome analysis has revealed increased expression of stress indicators (CANP and POSTN) in vitiligo skin, especially the oxidative stress marker CANP, which is consistent with (although not directly confirm) the oxidative stress theory of vitiligo pathogenesis [74]. The current study, combined with previously reported abnormal gene expression profile of melanocytes isolated from vitiligo skin [29], [30], [31], [32], raised the speculation that aberrant expression of stress markers on melanocytes (either as a result of intrinsic functional defect or external stimuli) may recruit and activate NK cells in the skin microenvironment, which may play an important role in the pathogenic process of vitiligo. CLEC2B gene, which encodes an activating ligand of the NK cells, is also found in our study to be up-regulated in vitiligo skin. This gene is mainly expressed by monocytes and macrophages, and can activate NK cells by binding to the NK cell-activating receptor NKp80 [75], [76], [77]. Since CLEC2B can be up-regulated by toll-like receptor stimulation [76], and topical imiquimod (an activator of the toll-like receptors) has been shown to induce vitiligo [78], [79], [80], we speculate that the increased expression of CLEC2B is a reflection of the activated innate immune system in vitiligo skin.

In addition to increased NK cells, this study revealed the presence of cytotoxic T cells in both vitiligo LS and NLS (**Figure 3**), whereas previous reports documented that these cells were present in the lesions and at the advancing borders of the lesions [28], [45]. Although our microarray analysis has also found increased expression of genes related to the Th1 pathway (including IFN-γ and IL-12), the Th17 pathway (IL-6), as well as genes associated with inflammation in general in vitiligo skin, they did not meet our strict cut-off criteria (2 or more fold changed with p<0.05 with Bonferonni Correction) and thus were not shown. It is currently unknown whether innate immune activation in vitiligo is a consequence of adaptive immune reaction to the melanocytes, or a contributing factor leading to the activation of adaptive immune reaction. More research is needed in the future to clarify this issue.

To our knowledge, this is the first genomic expression analysis on the skin of patients with vitiligo. Our results strengthened the notion that immunity and inflammation play important roles in vitiligo pathogenesis. Moreover, our study specifically highlights the potential pathogenic role of the aberrantly heightened innate immunity, especially NK cells, in the local microenvironment of melanocytes in vitiligo skin. Further studies in the future are needed to verify if increased NK cells and heightened innate immunity are the direct cause of melanocyte death in vitiligo. Finally, the observation of activated innate immunity in the normal appearing non-lesional skin far away from the lesional border suggests that therapeutically targeting the activated innate immune responses in both lesional and non-lesional vitligo skin may represent a viable approach for developing vitiligo therapies in the future.
