# Correction: Transcriptome Analysis Reveals Markers of Aberrantly Activated Innate Immunity in Vitiligo Lesional and Non-Lesional Skin

**DOI:** 10.1371/annotation/fbf9e317-e3ab-4f9a-b317-3de6abff890f

**Published:** 2013-07-15

**Authors:** Richard Yu, Raewyn Broady, Yuanshen Huang, Yang Wang, Jie Yu, Min Gao, Megan Levings, Shencai Wei, Shengquan Zhang, Aie Xu, Mingwan Su, Jan Dutz, Xuejun Zhang, Youwen Zhou

The number of the grant from the National Natural Science Foundation of China is incorrect. The correct number is 81171506. 

